# Characterizing the epithelial–mesenchymal transition status of circulating tumor cells in head and neck squamous cell carcinoma

**DOI:** 10.1002/hed.27167

**Published:** 2022-08-05

**Authors:** Karl Payne, Jill Brooks, Nikolaos Batis, Graham Taylor, Paul Nankivell, Hisham Mehanna

**Affiliations:** ^1^ Institute of Head and Neck Studies and Education, Institute of Cancer and Genomic Sciences University of Birmingham Birmingham UK; ^2^ Institute of Immunology and Immunotherapy University of Birmingham Birmingham UK

**Keywords:** biomarker, CTC, EMT, HNSCC, liquid biopsy

## Abstract

**Background:**

Circulating tumor cells (CTCs), in particular those undergoing an epithelial–mesenchymal transition (EMT), are a promising source of biomarkers in head and neck squamous cell carcinoma (HNSCC). Our aim was to validate a protocol using microfluidic enrichment (Parsortix platform) with flow‐cytometry CTC characterization.

**Method:**

Blood samples from 20 treatment naïve HNSCC patients underwent Parsortix enrichment and flow cytometry analysis to quantify CTCs and identify epithelial or EMT subgroups—correlated to clinical outcomes and EMT gene‐expression in tumor tissue.

**Results:**

CTCs were detected in 65% of patients (mean count 4 CTCs/ml). CTCs correlated with advanced disease (*p* = 0.0121), but not T or N classification. Epithelial or EMT CTCs did not correlate with progression‐free or overall survival. Tumor mesenchymal gene‐expression did not correlate with CTC EMT expression (*p* = 0.347).

**Discussion:**

Microfluidic enrichment and flow cytometry successfully characterizes EMT CTCs in HNSCC. The lack of association between tumor and CTC EMT profile suggests CTCs may undergo an adaptive EMT in response to stimuli within the circulation.

## 1 INTRODUCTION

Circulating tumor cells (CTCs) are one compartment of a liquid biopsy and are well established as a potential source of biomarkers in HNSCC.^1^, ^2^, ^3^, ^4^, ^5^ The transition of tumor cells from an epithelial to mesenchymal phenotype (EMT) is a key step in cancer progression, metastasis, and resistance to treatment.^6^ In HNSCC, EMT pathways are well described^7^ with evidence that tumors with mesenchymal‐like profiles have a poorer prognosis with increased risk of recurrence.^8^ Analysis of EMT‐related gene/protein expression in CTCs has therefore garnered particular interest and, as seen in tumor tissues, CTC EMT has been linked to poor clinical outcomes.^9^, ^10^

However, the optimal method of CTC enrichment remains unknown—comparing marker‐dependent methods that select a potentially biased population of CTCs based upon epithelial markers, versus methods that are marker‐independent and potentially able to select more diverse CTCs.^11^ Removing the inherent selection bias of marker‐dependent protocols, for example utilizing microfluidic enrichment, allows the isolation and analysis of CTCs with greater phenotypic diversity and the discovery of novel entities.^12^ Furthermore, the relationship between tumor and CTC protein expression, including EMT status, is under investigated. Here we validate a marker‐independent microfluidic CTC enrichment protocol combined with flow cytometry analysis to accurately quantify epithelial and EMT CTCs in HNSCC patient blood samples. Furthermore, their prognostic significance and relationship between tumor and CTC EMT status are examined.

## 2 METHODS

### 2.1 Blood sample processing

Nine milliliter peripheral blood samples were obtained from 20 treatment naïve HNSCC patients at time of curative surgery. Patients were recruited with written informed consent between October 2019 and March 2020 from a single institution (Queen Elizabeth Hospital, Birmingham, UK) through the ethically approved Accelerated2 sample collection platform (REC ref: 16/NW/0265). Blood was drawn directly into Transfix (Cytomark Ltd., Buckingham, UK) blood collection tubes, stored at 4°C and processed 24 h later using the Parsortix (Angle Plc, Surrey, UK) microfluidic CTC enrichment platform.^13^ In brief, the Parsortix platform is a device which pressurizes whole blood at 99 bar through a tiered microfluidic cassette, designed to capture cells based upon size (larger than 6.5 μm diameter) and compressibility/deformability characteristics.

**FIGURE 1:**
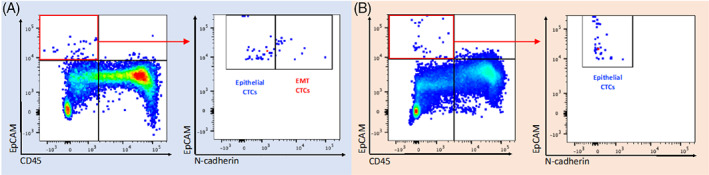
Identification of epithelial and mesenchymal CTCs. Results of Parsortix enrichment and flow cytometry analysis of blood from two HNSCC patients (panel A and B, respectively). CD45 high EpCAM low immune cells (bottom right quadrant in left hand plot) were used as a negative control to set boundaries for the quadrant gate allowing identification of EpCAM high, CD45 low CTCs. The CTC population in the red box was selected and levels of EpCAM and N‐cadherin assessed (right hand plot in panels A and B) to identify epithelial and EMT CTC phenotypic subgroups (gate boundaries set using positive control compensation bead data). Blood from patient A contained epithelial CTCs and EMT CTCs while blood from patient B contained only the former [Color figure can be viewed at wileyonlinelibrary.com]

Enriched CTCs were then analyzed by flow cytometry. The antibody panel was previously validated for use on Transfix fixed cells using HNSCC cell lines (FaDu and CAL27) and PBMC positive controls for the following markers: EpCAM (epithelial), N‐cadherin (mesenchymal), and CD45 (pan‐leukocyte).^14^ DAPI was used to stain nuclear DNA. We defined a CTC as a nucleated epithelial marker positive cell, which was negative for the pan‐leukocyte marker CD45. Parsortix enriched samples were analyzed using a three laser Becton Dickinson (BD) LSRII flow cytometer. Versacomp beads (Beckman Coulter, Brea, CA) were used to perform spectral compensation. Data analysis was performed using FlowJo version 10 (BD Biosciences, San Jose, CA). Flow cytometry data underwent standard clean‐up gating, using forward versus side scatter plots to remove debris and doublets. The CD45 high PBMC population was used as an internal negative control for EpCAM expression. Events that were EpCAM high and CD45 low were labeled as “epithelial CTCs” and events that were EpCAM and N‐cadherin high and CD45 low were labeled as “EMT CTCs” (Figure 1). N‐cadherin positive control data were derived from previous cell line validation experiments. Final CTC counts were presented as cells per ml whole blood enriched.

### 2.2 Threshold of CTC detection in patients

Using the above methodology, we analyzed blood from five healthy donor (HD) controls to define a cut‐off threshold for CTC detection in patients with cancer. EpCAM high and CD45 low events were undetectable in two out of five patients and were present at a low frequency in three out of five HDs—at six, eight, and nine events per 9 ml blood sample. We therefore selected more than nine events per 9 ml blood sample (the highest frequency measured in HDs, corresponding to >1 CTC/ml blood) as a threshold of CTC positivity in patients. Patient samples with a CTC count below this threshold were classified as CTC negative. We attributed such “false‐positive CTC” events as likely originating from technical artifact from antibody staining or cytometry processing/data clean‐up gating. It should also be noted that low levels of circulating epithelial cells have been noted in patients without cancer, for example benign colon or liver disease.^15^, ^16^ Regardless, circulating epithelial cells are still proven as prognostic markers in patients with cancer; thus, we adhered to accepted CTC defining criteria from other reports. However, given we were analyzing 10–50 000 cells (the enriched cell output from the Parsortix device) this potential artefactual/false positive rate was very low, in the region of 0.02%.

### 2.3 Tumor tissue RNA sequencing

RNA from fresh frozen primary tumor tissue (tumor core) was extracted using the Qiagen RNeasy mini kit as per manufacturer's instructions. Quantitative mRNA expression analysis was undertaken using the Lexogen QuantSeq library preparation and Illumina Nextseq 500 platform. Data from RNA sequencing were analyzed using Bioconductor packages within R. Sequencing reads were trimmed to remove adapter contamination, polyA read through and low‐quality tails using the bbduk tool according to Lexogen guidelines. The trimmed reads were mapped to the GRCh38 (hg38) human genome and counted using STAR aligner (v2.5.2b).^17^ Normalization of read counts was performed using DESeq2.^18^ Using the HNSCC EMT signature proposed by Jung et al.,^8^ tumor transcript counts underwent comparative analysis and hierarchical clustering to identify “epithelial” and “mesenchymal” groups.

## 3 RESULTS

### 3.1 CTC count and tumor stage

Clinicopathological data and CTC count (cells/ml whole blood) are shown in Table 1. None of the patients had evidence of distant metastasis at diagnosis. Mean patient age was 65.6 years (range 24–84) with 65% (*n* = 13) male and 35% (*n* = 7) female. The majority of patients (13/20) presented with advanced (stage III/IV) disease, with an “oral” anatomical subsite predominance (11/20).

**TABLE 1:** Patient demographic and clinical data with CTC count

Pt no.	Age	Sex	T stage	N stage	Stage grouping	Tumor site	Epithelial CTC	EMT CTC	Total CTC
1	68	F	4	2	IV	Oral	2.6	2.1	4.7
2	72	M	2	1	III	Hypopharynx	3.9	1.8	5.7
3	62	M	x	2	III	Unknown	1.3	1.2	2.6
4	62	M	2	0	II	Oral	0	0	0
5	69	F	3	0	III	Oral	1.7	0.8	2.4
6	67	M	2	0	II	Oropharynx	4	0.4	4.4
7	57	F	2	0	II	Oral	0	0	0
8	50	M	4	2	IV	Oropharynx	1.6	1.2	2.8
9	73	F	4	0	IV	Oral	6.9	4.3	11.2
10	64	F	4	2	IV	Oral	2.9	1.1	4
11	68	M	2	0	II	Larynx	2.4	0	2.4
12	25	F	1	0	I	Oral	0	0	0
13	84	F	4	0	IV	Oral	3.8	0	3.8
14	61	M	2	0	II	Oral	0	0	0
15	79	M	4	0	IV	Larynx	0	0	0
16	54	M	4	3	IV	Larynx	0	0	0
17	65	M	2	0	II	Oral	0	0	0
18	76	M	4	2	IV	Larynx	1.8	1	2.8
19	58	M	4	0	IV	Oral	2.3	1.4	3.7
20	79	M	2	2	III	Oral	2	0	2

*Note*: Cell count of epithelial and EMT CTCs identified from a 9‐ml transfix BCT is shown as CTCs/ml whole blood.

CTCs were characterized into “epithelial” or “EMT” (expressing both epithelial and mesenchymal markers) phenotypic subgroups—based upon EpCAM and N‐cadherin expression. Sixty‐five percent (13/20) of patients were CTC positive. All 13 positive patients demonstrated epithelial CTCs and 10 of these also demonstrated EMT CTCs. Among CTC positive patients, the mean total cell count was 4 CTCs/ml blood (range 2–11.2).

**FIGURE 2:**
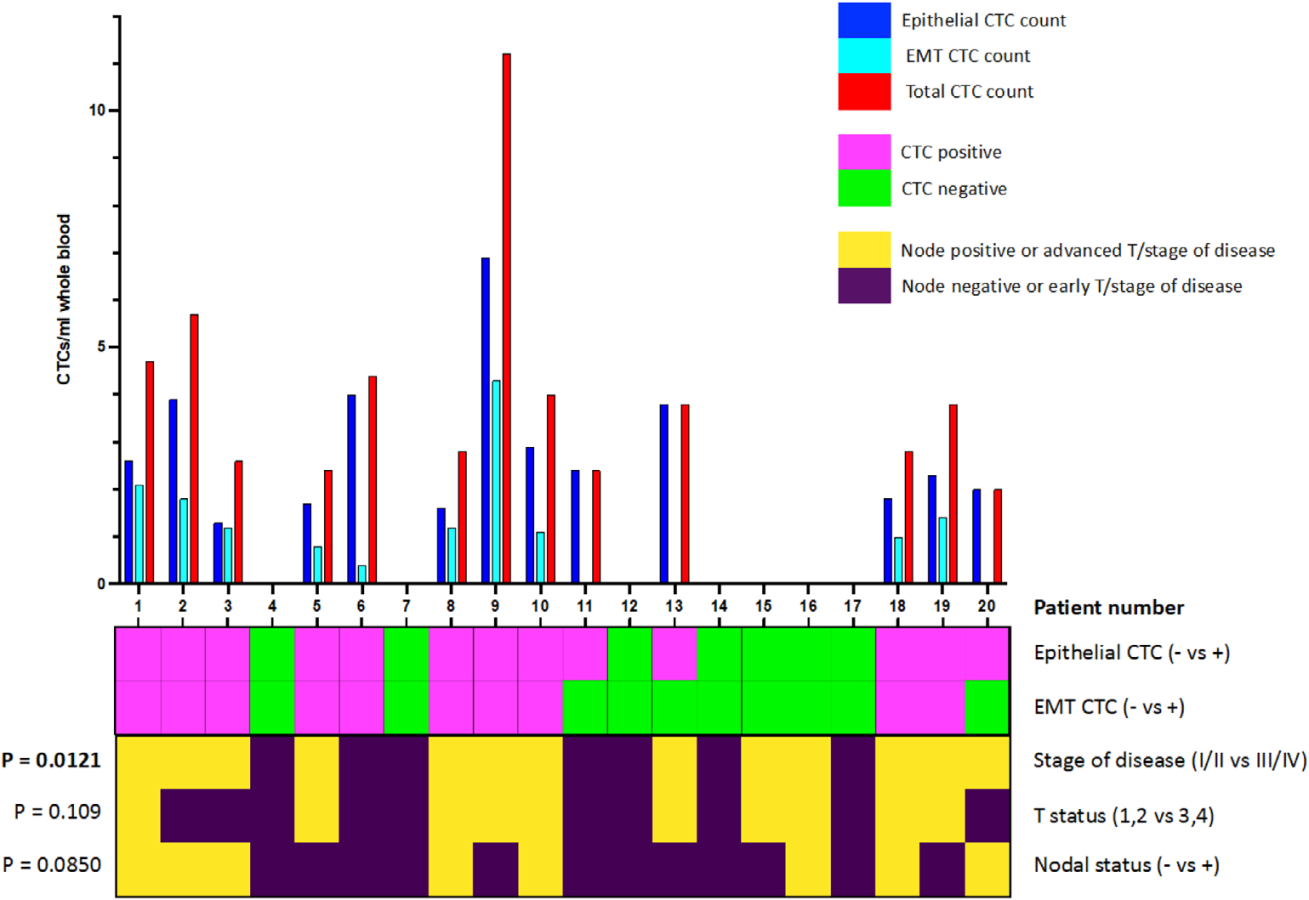
Patient CTC count and correlation with disease stage of disease. Bar chart (upper panel) displays number of epithelial, EMT, and total CTCs (per ml of whole blood) for 20 patients. Heatmap (lower panel) summarizes CTC detection (top two rows) and patient disease status (lower three rows). The *p* values show the results of chi squared tests of the relationship between CTC positivity (epithelial or EMT type) and disease stage, T status or N status [Color figure can be viewed at wileyonlinelibrary.com]

Comparing the presence of CTC phenotypic subgroups to clinical data, we observed that five of the seven CTC‐negative patients had early stage (I/II) disease and the presence of CTCs of any phenotype was significantly associated with advanced stage (III/IV) disease (chi‐square, *p* = 0.0121; Figure 2). Furthermore, EMT CTCs were almost exclusively observed in advanced stage disease patients (9/10). The presence of CTCs (of either phenotype) was not associated with T (1/2 vs. 3/4) or N (N0 vs. N^+ve^) classification (chi‐square, *p* = 0.109 and 0.0850, respectively; Figure 2). Multivariate analysis was performed using Poisson regression—modeling CTC phenotype count (epithelial and EMT) against T and N classification. Neither epithelial nor EMT CTC count could be fitted into a prediction model for T classification (*p* = 0.882 and 0.418, respectively) or N classification (*p* = 0.280 and 0.224, respectively). The ratio of EMT to epithelial CTCs was calculated and compared to total CTC count. These values were significantly correlated (Pearson's *R* = 0.564, *p* = 0.00958). Thus, as the total CTC count increased so did the fraction of EMT CTCs within the entire CTC population.

### 3.2 Pretreatment CTC count as a prognostic marker of patient survival

Median patient follow‐up time was 586 days (range 324–674). Of the 20 patients within the cohort, at the time of censoring—eight patients had died (five of which died due to disease progression and three due to other causes) and one patient was alive but with local recurrence. Of those patients who died disease progression—two had local recurrence, two had regional (cervical lymph node) metastases and one had distant metastases.

Concerning tumor pathological variables, the T classification of the tumor (stage 1/2 vs. 3/4) or overall stage of disease (early vs. late stage) did not correlate with overall survival (OS) or progression‐free survival (PFS). However, the presence of nodal metastases was significantly associated with OS and PFS (*p* = 0.0190 and 0.0219, respectively, Mantel‐Cox log‐rank).

**FIGURE 3:**
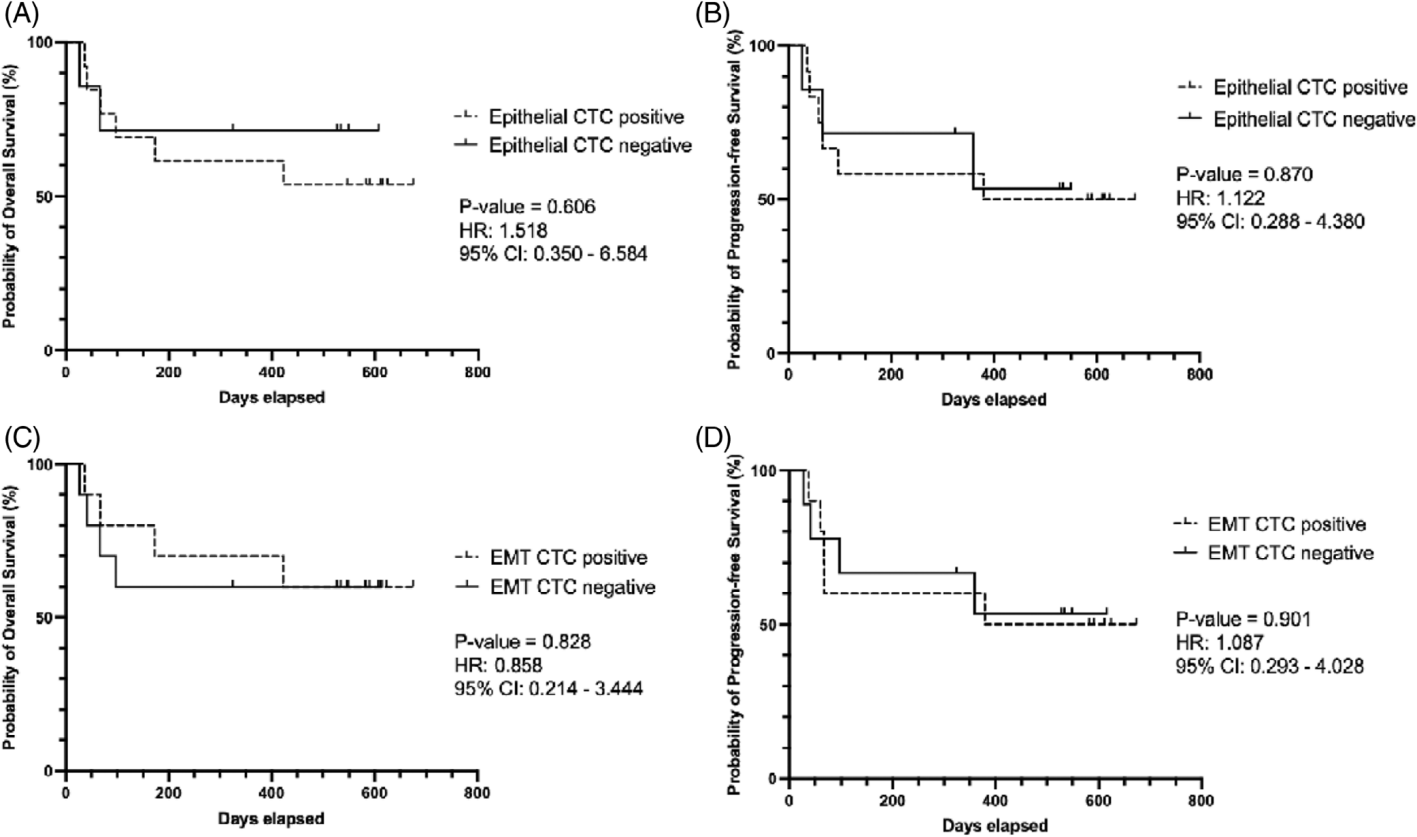
Relationship between CTC positivity and survival outcomes. Kaplan–Meier plots of overall (OS) and progression‐free survival (PFS) for epithelial (A, B) and EMT (C, D) CTC positive groups. Log‐rank (Mantel‐Cox) derived *p*‐value and hazard ratio reported. Neither epithelial nor EMT CTC presence correlated with OS or PFS

Regarding the prognostic utility of epithelial and/or EMT CTC presence in a pretreatment blood sample, neither the presence of epithelial nor EMT CTCs were significantly associated with OS nor PFS (Figure 3). In addition, we were unable to determine a cut‐off threshold of CTC count with prognostic significance for OS or PFS. Patients with epithelial CTCs had a decreased probability of OS (71% vs. 54%; Figure 3A), but not to a significant level. In addition, pretreatment CTC presence did not predict regional/distant metastasis during follow‐up (*p* = 0.640, chi‐square).

### 3.3 Comparison of tumor and CTC EMT profiles

**FIGURE 4:**
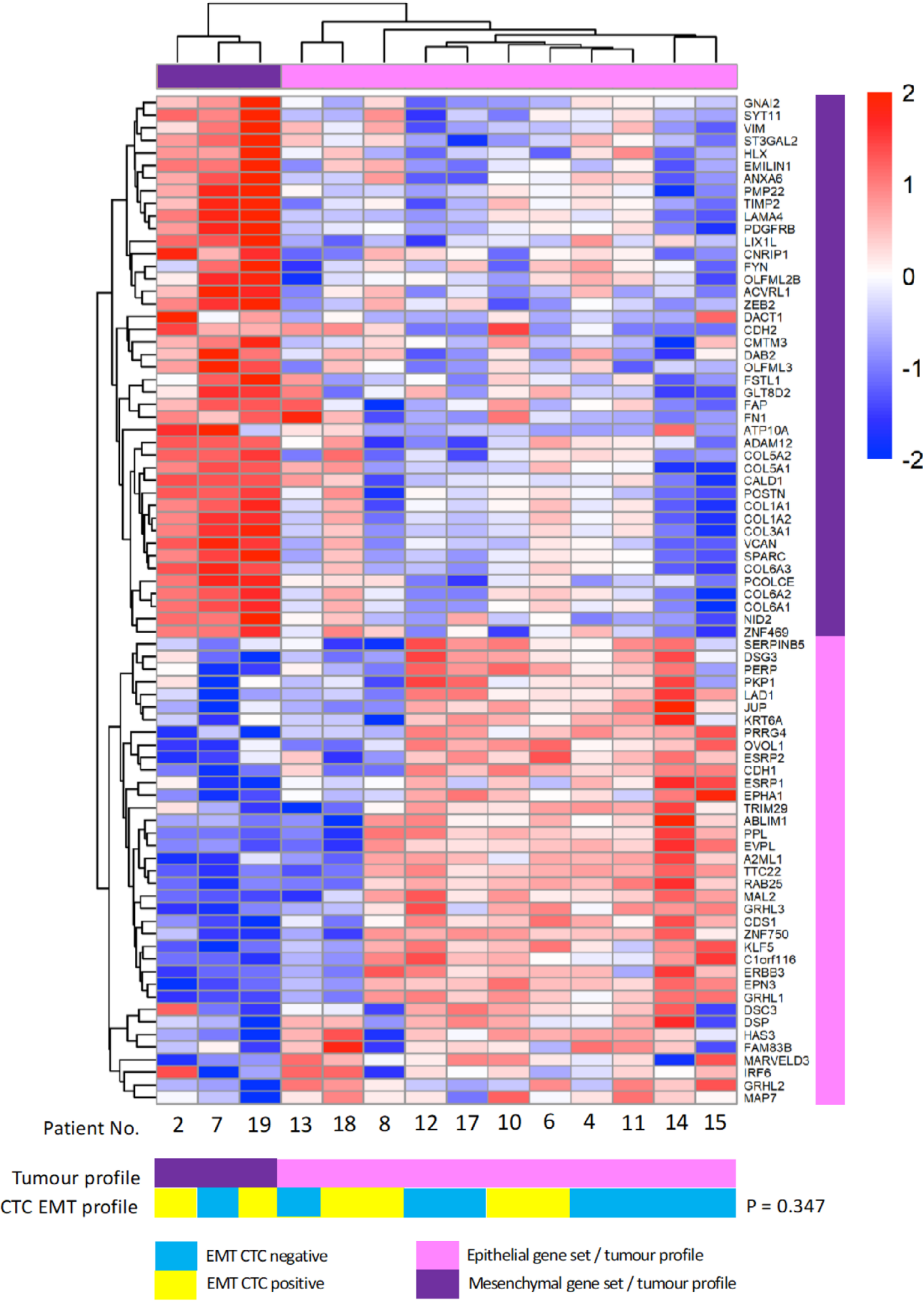
Blood CTC EMT profile does not correlate with tumor mesenchymal status. Heat map of gene expression (key denotes z score) in tumor samples from 14 HNSCC patients in our cohort. The genes that are shown correspond to a published 82 gene EMT signature. Hierarchical clustering divided patients into mesenchymal (*n* = 3) and epithelial (*n* = 11) groups. Tumor profile was compared to CTC EMT expression (below) with no significant association (chi‐square test) [Color figure can be viewed at wileyonlinelibrary.com]

We next examined how the presence of epithelial and EMT CTCs in the blood related to the mesenchymal status of the same patients' tumor tissue. Core tumor tissue samples were available from 14 patients within our cohort. Gene expression profiling was performed using quantitative mRNA expression analysis. To separate tumors into epithelial and mesenchymal groups, we utilized a previously validated HNSCC EMT gene signature of 38 epithelial and 44 mesenchymal associated genes.^8^ Clustering analysis revealed that of the 14 tumors sequenced, 3 demonstrated increased mesenchymal related gene expression while the remaining 11 exhibited decreased mesenchymal gene expression and/or increased epithelial related gene expression (Figure 4). The presence of EMT CTCs in the blood of these patients was not significantly associated with tumor mesenchymal status (chi‐square, *p* = 0.347; Figure 4). In addition, the tumor mesenchymal status was not associated with stage of disease or nodal metastasis (chi‐square, *p* = 0.515 and 0.837, respectively).

**FIGURE 5:**
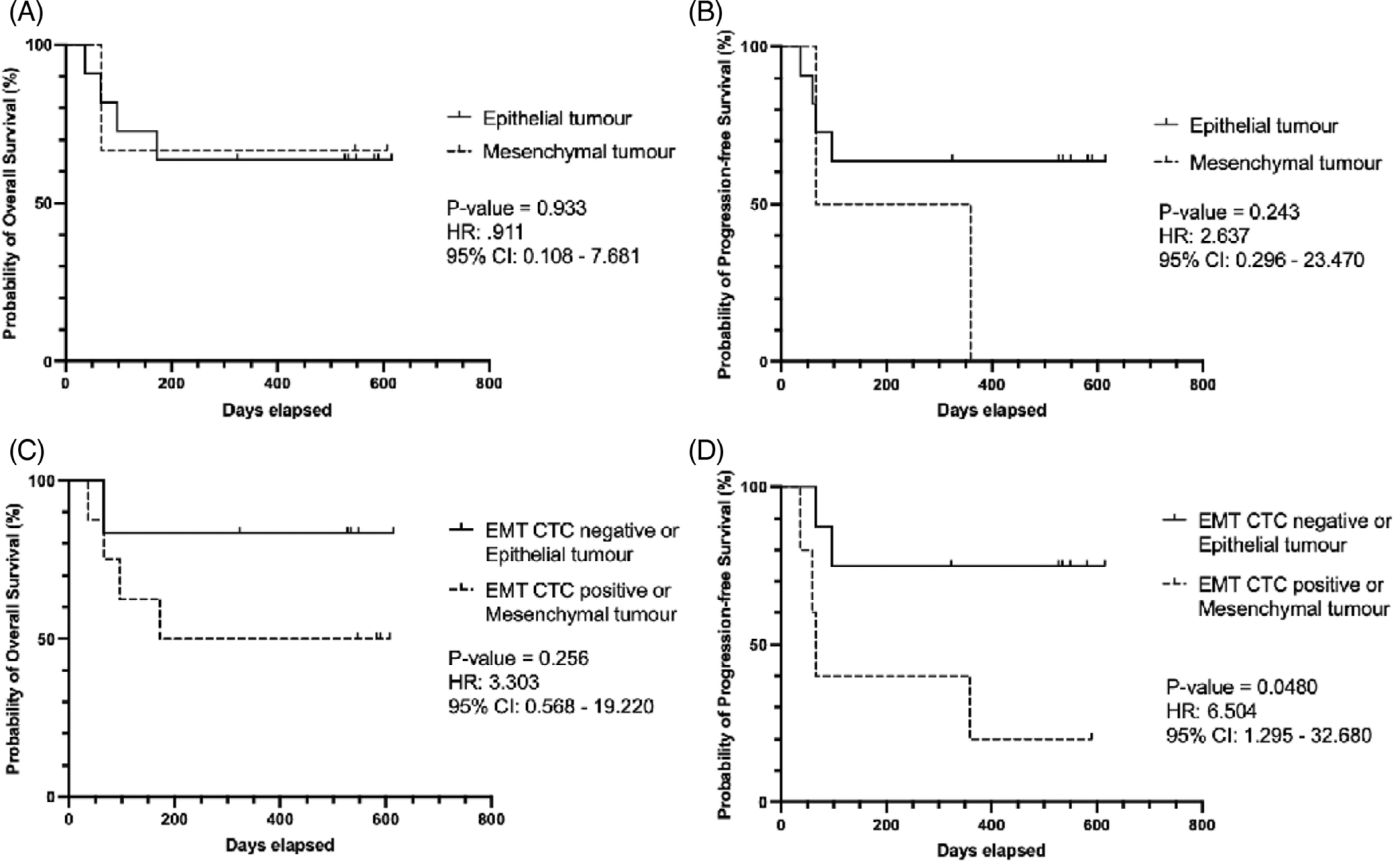
Mesenchymal expression in tumor and CTC results in significantly worse progression‐free survival. Kaplan–Meier plots of overall (OS) and progression‐free survival (PFS) for tumor status (A, B) and mesenchymal profile in CTC or tumor samples (C, D). Log‐rank (Mantel‐Cox) derived *p*‐value and hazard ratio reported. Patients with a mesenchymal profile in either tumor or CTCs had significantly worse PFS (D, *p* = 0.0480)

Although tumor mesenchymal status did not significantly associate with OS or PFS, there was a trend of decreased PFS within mesenchymal profile tumors (Figure 5A,B). In addition, all patients with a mesenchymal tumor profile suffered recurrence. We therefore investigated if combining information of tumor and CTC mesenchymal status could provide improved prognostic stratification in our cohort. Patients with a mesenchymal profile in *either* tumor *or* CTCs had significantly worse PFS, with a HR of 6.5 and a difference in probability of PFS of 75% vs. 20% at 600 days follow‐up (*p* = 0.0480, Mantel‐Cox log‐rank; Figure 5D). There was also a trend towards decreased OS in the mesenchymal group, with a HR of 3.3, but this was not statistically significant (Figure 5C).

## 4 DISCUSSION

Previous reports have discussed the use of flow cytometry and “immunomagnetic” epithelial marker‐based cell enrichment to detect and quantify CTCs in multiple cancer types,^19^, ^20^, ^21^ including HNSCC.^22^ We provide primary evidence for the utility of Parsortix microfluidic “cell size dependent” enrichment combined with flow cytometry to successfully quantify and characterize CTC phenotypic subgroups. The utility of microfluidic CTC enrichment is now well described and validated in several cancer types across multiple commercially available platforms.^12^, ^23^ As discussed, the primary advantage of this method is its “marker‐independent” approach which removes potential cell selection bias. In addition, evidence suggests that microfluidic enrichment has been shown as superior to leukocyte size‐based enrichment (i.e. density gradient centrifugation).^14^, ^24^ While the “marker‐dependent” CellSearch platform was the first FDA approved platform, recent evidence in HNSCC and breast cancer has demonstrated the benefit of Parsortix microfluidic enrichment to detect phenotypically heterogeneous CTCs.^25^, ^26^ We accept that the true benefit of microfluidic enrichment is not fully realized in our analysis; only a limited number of markers per cell could be measured due to the limitations of spectral overlap in conventional flow cytometry. However, new multispectral or mass cytometry platforms now allow much larger antibody panels, able to detect 30–40 markers, to be utilized providing a viable means of multiparameter single‐CTC proteomic analysis. Our protocol of Parsortix enrichment and minimal CTC handling via in‐cassette staining provides proof‐of‐concept and would be directly applicable to these assays.

Using CTCs as diagnostic biomarkers of disease burden and prognostic biomarkers for survival outcomes has been reported by multiple groups.^1^ In our cohort, 65% (13/20) demonstrated evidence of CTCs, which is comparable to previous data in HNSCC.^27^ We observed no relationship between CTC count or presence and individual clinicopathological variables. However, the presence of CTCs positively associated with advanced (III/IV) overall stage of disease (*p* = 0.0121). Previous reports have demonstrated an N classification of ≥2 is associated with CTC presence^22^—in our cohort it was noteworthy that six out of seven patients with an N classification ≥2 were CTC positive. It would seem logical from a biological and mechanistic standpoint that a tumor with greater burden of disease and metastatic potential, that is, larger and with nodal metastases, would produce CTCs or increased numbers of CTCs. However, published data are equivocal in this regard and meta‐analyses of HNSCC have not been able to definitively correlate TNM or overall stage of disease with CTC presence.^28^, ^29^ While the majority of published data remains heterogeneous and from small cohorts, in general the presence of CTCs appears to correlate with increased rates of recurrence and decreased survival, although current evidence is far from conclusive.^27^, ^29^ While we observed a decreased probability of OS at 2‐year follow‐up in epithelial CTC positive patients, we were unable to demonstrate significant associations between epithelial or EMT CTC presence and clinical outcomes. In addition, regional/distant metastasis during follow‐up did not correlate with pretreatment CTC positivity.

Much of the biology of CTCs remains poorly understood and largely derived from in vitro and/or in vivo animal models^30^ with several key questions still unanswered. Primarily—*How do CTCs get into the circulation and how do they change once they are there*? The mechanism of CTC intravasation is thought to be a combination of passive and active migration, with the latter occurring via an EMT to an invasive and motile mesenchymal phenotype.^31^ The presence of EMT CTCs in HNSCC has been suggested to be a marker of R/M and decreased survival.^9^, ^10^ While we were unable to correlate an EMT CTC profile with survival outcomes, we did observe that EMT CTCs were seen almost exclusively in advanced stage disease patients. A strong trend was observed between the ratio of EMT to epithelial CTCs and N‐stage (Pearson *R* = 0.4125, *p* = 0.0642). Thus, patients with a greater burden of nodal metastasis exhibited an increased likelihood of a mesenchymal profile within their CTC population, further highlighting the metastatic potential of this CTC subgroup.

The presence of CTCs with heterogeneous epithelial and EMT phenotypes raises the question of whether this profile is representative of the tumor and thus is a by‐product of EMT required for intravasation, or is an adaptation required for CTCs to survive within the circulation. Very few studies have compared CTC and tumor expression profiles. Studies comparing CTC EMT profiles to tumor pathology‐based prognostic markers, such as vascular invasion, have not revealed positive correlations.^32^ In HNSCC specifically, while studies have compared tumor and CTC immune marker expression (reporting no correlation^33^), to‐date no study in HNSCC has described the relationship between tumor and CTC mesenchymal status. We report that the EMT profile of CTCs and tumor mesenchymal status was not concordant, although this assertion should be tempered by a relatively small pilot patient cohort. Nevertheless, it raises further questions as to the potential EMT plasticity of CTCs^34^—the concept that tumor cells may enter a dynamic transitional state upon entry into the circulation. For example, do CTCs shift between epithelial and mesenchymal phenotypes as an adaptive response to various stimuli, independent of tumor mesenchymal status? We note that in our data EMT CTCs were only observed in the presence of epithelial CTCs, consistent with an EMT potentially being an adaptive function of a subset of epithelial tumor cells surviving within the circulation.

We accept that this study presents a small pilot cohort, chosen to evaluate our enrichment and characterization protocol. Thus, possible explanations for the lack of association with clinicopathological and outcome variables, as observed in other studies, may be attributed to a low patient number, which for the purposes of “proof‐of‐principle” was not subject to a formal power calculation. Larger cohort clinical studies would be required to validate our protocol and explore potential correlations between clinical outcomes and tumor phenotype.

In summary, we demonstrate that microfluidic CTC enrichment and flow cytometry‐based characterization is able to accurately quantify CTCs and identify those cell subgroups undergoing an EMT. If, as our study suggests, CTC EMT status is independent to that of the tumor, then novel CTC EMT biomarkers may hold translational potential as prognostic and/or predictive biomarkers, beyond those obtained from tumor characterization. However, evidence continues to grow that EMT cannot be described based upon a single pathway only—the interaction between EMT transcription factors (e.g., Twist and Snail) and mesenchymal cell surface markers is both complex and dynamic.^35^ Therefore, strategies that use single marker identification may underestimate CTC burden.^36^ Future studies should seek to align Parsortix microfluidic enrichment with single‐CTC characterization, both transcriptomic and proteomic. Only once a standardized protocol has been validated for technical feasibility can large cohort clinical studies be designed with a translational viewpoint. Furthermore, clinical trials should seek to answer the question regarding the predictive utility of a CTC liquid biopsy (i.e., CTC immune checkpoint expression directing personalized treatment^37^), especially in recurrent/metastatic patients not amenable to repeat tissue biopsies.

## CONFLICT OF INTEREST

The authors declare that there is no conflict of interest that could be perceived as prejudicing the impartiality of the research reported.

## ETHICS STATEMENT

Blood and tissue samples were collected with patient consent as part of the ethically approved Accelerated2 HNSCC sample collection study (REC ref: 16/NW/0265).

## Data Availability

The data that support the findings of this study are available from the corresponding author upon reasonable request.
